# Spectral, thermal, molecular modeling and biological studies on mono- and binuclear complexes derived from oxalo bis(2,3-butanedionehydrazone)

**DOI:** 10.1186/s13065-015-0135-y

**Published:** 2015-12-29

**Authors:** Ahmed El-Asmy, Bakir Jeragh, Mayada Ali

**Affiliations:** Chemistry Department, Faculty of Science, Kuwait University, 5969, Kuwait city, Safat 1360, Kuwait

**Keywords:** Hydrazones, Spectra, TGA, Biological activity, X-ray crystallography

## Abstract

**Background:**

Hydrazones and their metal complexes were heavily studied due to their pharmacological applications such as antimicrobial, anticonvulsant analgesic, anti-inflammatory and anti-cancer agents. This work aims to synthesize and characterize novel complexes of VO^2+^, Co^2+^, Ni^2+^, Cu^2+^, Zn^2+^, Zr^4+^and Pd^2+^ ions with oxalo bis(2,3-butanedione-hydrazone). Single crystals of the ligand have been grown and analyzed.

**Results:**

Oxalo bis(2,3-butanedionehydrazone) [OBH] has a monoclinic crystal with P 1 21/n 1 space group. The VO^2+^, Co^2+^, Ni^2+^, Cu^2+^, Zn^2+^, Zr^4+^ and Pd^2+^ complexes have the formulas: [VO(OBH–H)_2_]·H_2_O, [Co(OBH)_2_Cl]Cl·½EtOH, [Ni_2_(OBH)Cl_4_]·H_2_O·EtOH, [Cu(OBH)_2_Cl_2_]·2H_2_O, [Zn(OBH–H)_2_], [Zr(OBH)Cl_4_]·2H_2_O, and [Pd_2_(OBH)(H_2_O)_2_Cl_4_]·2H_2_O. All complexes are nonelectrolytes except [Co(OBH)_2_Cl]Cl·½EtOH. OBH ligates as: neutral tetradentate (NNOO) in the Ni^2+^ and Pd^2+^ complexes; neutral bidentate (OO) in [Co(OBH)_2_Cl]Cl·½EtOH, [Zr(OBH)Cl_4_]·2H_2_O and [Cu(OBH)_2_Cl_2_]·2H_2_O and monobasic bidentate (OO) in the Zn^2+^ and VO^2+^ complexes. The NMR (^1^H and ^13^C) spectra support these data. The results proved a tetrahedral for the Zn^2+^ complex; square-planar for Pd^2+^; mixed stereochemistry for Ni^2+^; square-pyramid for Co^2+^ and VO^2+^ and octahedral for Cu^2+^ and Zr^4+^ complexes. The TGA revealed the outer and inner solvents as well as the residual part. The molecular modeling of [Ni_2_(OBH)Cl_4_]·H_2_O·EtOH and [Co(OBH)_2_Cl]Cl·½EtOH are drawn and their molecular parameters proved that the presence of two metals stabilized the complex more than the mono metal. The complexes have variable activities against some bacteria and fungi. [Zr(OBH)Cl_4_]·2H_2_O has the highest activity. [Co(OBH)_2_Cl]Cl·½EtOH has more activity against *Fusarium.*

**Conclusion:**

Oxalo bis(2,3-butanedionehydrazone) structure was proved by X-ray crystallography. It coordinates with some transition metal ions as neutral bidentate; mononegative bidentate and neutral tetradentate. The complexes have tetrahedral, square-planar and/or octahedral structures. The VO^2+^ and Co^2+^ complexes have square-pyramid structure. [Cu(OBH)_2_Cl_2_]·2H_2_O and [Ni_2_(OBH)Cl_4_]·H_2_O·EtOH decomposed to their oxides while [VO(OBH–H)_2_]·H_2_O to vanadium. The energies obtained from molecular modeling calculation for [Ni_2_(OBH)Cl_4_]·H_2_O·EtOH are less than those for [Co(OBH)_2_Cl]Cl·½EtOH indicating the two metals stabilized the complex more than mono metal. The Co(II) complex is polar molecule while the Ni(II) is non-polar.

**Graphical abstract:**

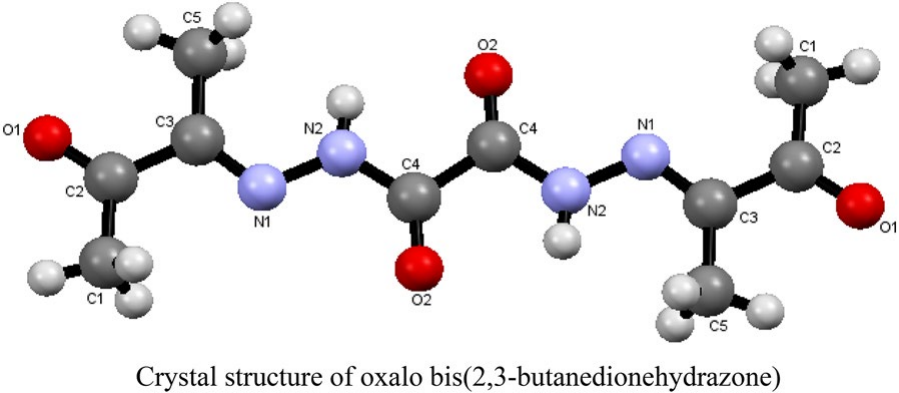

**Electronic supplementary material:**

The online version of this article (doi:10.1186/s13065-015-0135-y) contains supplementary material, which is available to authorized users.

## Background

Hydrazones and their metal complexes are heavily studied compounds which have many pharmacological applications such as antimicrobial, anticonvulsant analgesic, anti-inflammatory and anti-cancer agents. Acetylpyridine and benzoylpyridine hydrazones were used as reagents against brain tumor and are highly cytotoxic to glioma cells [1]. Interest has been focused on hydrazone complexes to study their anti-parasitic, fungicidal and bactericidal properties [2, 3]. 2,3-Butanedione monoxime possessed cardio protective properties related to the inhibition of cross-bridge force development [4]. Heterocyclic compounds containing nitrogen have much attention due to their activity as antitumor, anti-inflammation, anti-pyretic, antiviral, anti-microbial, insecticides and fungicides [5–7]. Isonicotinyl hydrazone complexes of 2-acetylpyridine, pyrrolyl-2-carboxaldehyde, 2,5-dihydroxy-acetophenone, N-isonicotinamido-furfuraldimine, 2-thiophenecarbonyl and 3-(N-methyl)-isatin were reported [8–12]. The Ni(II) and Cu(II) complexes of 2,3-butanedione bis(N(3)substituted-thiosemicarbazones) were studied and some of these compounds were solved by x-ray crystallography [13]. The crystal structures of [Cu(HxPip-2H)] (HxPip = 3,4-hexanedione bis(3-piperidylthiosemicarbazone) and [Cu(HxHexim-2H)] (HxHexim = 3,4-hexanedione bis(3-hexa-methyleneiminylthiosemicarbazone) were solved having a square-planar geometry [14]. Binuclear complexes of VO^2+^, Co^2+^, Ni^2+^, Cu^2+^ and Zn^2+^ with oxalyl bis(diacetylmonoximehydrazone) were characterized as 2:2 (M:L) and an octahedral geometry for VO^2+^, tetrahedral for Zn^2+^ and square-planar for the rest complexes were proposed [15]. On continuation to our work on bis(hydrazones) and their complexes [16, 17], this work aims to synthesize and characterize novel complexes of VO^2+^, Co^2+^, Ni^2+^, Cu^2+^, Zn^2+^, Zr^4+^and Pd^2+^ ions with oxalo bis(2,3-butanedione-hydrazone). Single crystals for the ligand have been grown and analyzed. Trials to grow crystals for the complexes were failed, so molecular modeling for the Co(II) and Ni(II) complexes were done.

### Experimental

VOSO_4_·2H_2_O, CoCl_2_·6H_2_O, NiCl_2_·6H_2_O, CuCl_2_·2H_2_O, ZnCl_2_·2H_2_O, K_2_PdCl_4_ and ZrCl_4_, diethyl oxalate, hydrazine hydrate, 2,3-butanedione, ethanol, diethyl ether, DMF and DMSO were obtained from the BDH chemicals.

### Synthesis of oxalo bis(2,3-butanedionehydrazone) [OBH]

OBH was prepared by heating under reflux a suspension (6 g, 0.05 mol) of oxalic acid dihydrazide in 50 mL EtOH and 8.6 ml (0.1 mol) of 2,3-butanedione on a heating mantle for 10 h. The precipitate thus formed was filtered off, recrystallized from ethanol and finally dried. It was characterized by elemental analysis and spectral studies. The ^1^H NMR spectrum of the ligand showed signals at δ = 11.924 (s, 2H) and 2.129 (s, 6H) ppm for the NH and CH_3_ protons. Its ^13^C NMR showed peaks at 196.65, 167.58, 148.81 and 23.90 ppm for (C=O)_ketonic_ (C=O)_amidic_, C=N and CH_3_, respectively.

### Preparation of the metal complexes

The metal complexes were prepared by reacting calculated amounts corresponding to 2:1 ratio [M:L] in 50 mL EtOH and the mixture was heated under reflux for 6–8 h. In the preparation of VO^2+^ complex, 0.1 g of sodium acetate was added to raise the pH (~8) and precipitating the complex. The formed precipitates were filtered off, washed with hot water, hot ethanol and diethyl ether and finally dried in a vacuum desiccator over anhydrous silica gel. Attempts to grow single crystals for the complexes were done but unsuccessful.

### Analysis and equipment

Carbon, hydrogen and nitrogen content of the compounds were determined at the Microanalytical Unit (Varian Micro V1.5.8, CHNS Mode, 15073036) of Kuwait University. The metal content was determined using ICP-OES GBC Quantium Sequential at Kuwait University. The mass spectra were recorded on a GC–MS Thermo-DFS (BG_FAB) mass spectrometer. The melting points were measured on a Griffin melting point apparatus. The conductance for 10^−3^ mol L^−1^ DMSO solution of the compounds was measured on Orion 3 STAB Conductivity Bridge. The IR spectra were recorded as KBr discs on a FT/IR-6300 type A (400–4000 cm^−1^). The electronic spectra of the complexes were recorded on a Cary 5 UV–vis spectrophotometer, varian (200–900 nm). The ^1^H NMR spectra of the ligand and the diamagnetic complexes were recorded in DMSO-d6, on a Bruker WP 200 SY Spectrometer (400 MHz) at room temperature using tetramethylsilane (TMS) as an external standard. The magnetic measurements were carried out on a Johnson-Matthey magnetic balance, UK. The TGA thermograms were recorded (25–800 °C) on a Shimadzu TGA-60; the nitrogen flow and heating rate were 50 ml/min and 10 °C min^−1^, respectively. The X-ray single crystal diffraction data were collected on a Rigaku R-Axis Rapid diffractometer using filtered Mo-K α-radiation. The structure was solved by the direct methods and expanded using Fourier techniques at Kuwait University. The ligand and its complexes were investigated for antimicrobial activity against *Bacillus, Aspergillus, Escherichia coli, Pennicillium* and *Fusarium* as reported earlier [15]. All molecular calculations were carried out by HyperChem 7.51 software package. The molecular geometry of the Co^2+^ and Ni^2+^ complexes are first optimized at molecular mechanics (MM+) level. Semi empirical method PM3 is then used for optimizing the full geometry of the system using Polak–Ribiere (conjugate gradient) algorithm and Unrestricted Hartee-Fock (UHF) is employed keeping RMS gradient of 0.01 kcal/Å mol.

## Results and discussion

### Crystal analysis of OBH

The crystal structure of OBH is shown in Structure 1. Its refinement data are summarized in Table 1 while the bond lengths and bond angles are presented in Table 2. OBH was crystalized as monoclinic system and P 121/n1 space group with molecular weight of 254.25. The N_1_–C_3_, O_1_–C_2_ and O_2_–C_4_ distances are 1.283(3), 1.210(3) and 1.206 Å, respectively, indicating true double bond; the amidic carbonyl has value slightly higher than the ketonic carbonyl. The N_2_–C_4_ and N_1_–N_2_ are 1.351(4) and 1.381 Å indicating single bonds. All bond angles are between 115 and 127 and 109.5° meaning the trigonal and tetrahedral geometries with sp^2^ and sp^3^ hybridization. The presence of lone pair of electrons on N_1_ in C_3_N_1_N_2_ reduces the angle from 120° to 115.7°. The bond angle of N_2_–C_4_–C_4_ reduces to 110.6°, in consistent with some distortion, while that of O_2_–C_4_–N_2_ increases to 126.9° due to the existence of two more electronegative atoms (O atoms).

**Structure 1:**
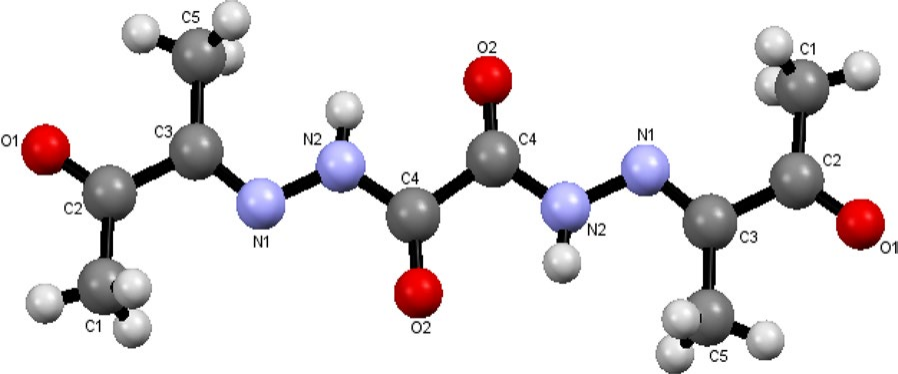
Crystal structure of oxalo bis(2,3-butanedionehydrazone)

**Table 1:** Crystallographic data for OBH crystal

Identification code	OBH
Chemical formula	C_10_H_14_N_4_O_4_
Formula weight	254.25
Temperature	296 (2) K
Wavelength	1.54178 Å
Crystal size	0.020 × 0.120 × 0.230 mm
Crystal habit	Clear light colorless flakes
Crystal system	Monoclinic
Space group	P 1 21/n 1
Unit cell dimensions	a = 6.3630 (5) Å, α = 90^o^ b = 4.6609 (4) Å, β = 91.37^o^ c = 20.7562 (19) Å, γ = 90^o^
Volume	615.40 (9) Å^3^
Z	2
Density (calculated)	1.372 g/cm^3^
Absorption coefficient	0.915 mm^−1^
F (000)	268

**Table 2:** Bond lengths and bond angles of OBH

Bond	Length	Bond	Length
O1–C2I	1.210 (3)	O2–C4	1.206 (3)
N1–C3	1.283 (3)	N1–N2	1.381 (3)
N2–C4	1.351 (4)	N2–H7	0.86
C1–C2	1.479 (4)	C1–H1	0.96
C1–H2	0.96	C1–H3	0.96
C2–C3	1.506 (4)	C3–C5	1.486 (4)
C4–C4#1	1.529 (6)	C5–H5	0.96
C5–H4	0.96	C5–H6	0.96

Bond	Angle (°)	Bond	Angle (°)
C3–N1–N2	115.7 (2)	C4–N2–N1	120.6 (2)
C4–N2–H7	119.7	N1–N2–H7	119.7
C2–C1–H1	109.5	C2–C1–H2	109.5
H1–C1–H2	109.5	C2–C1–H3	109.5
H1–C1–H3	109.5	H2–C1–H3	109.5
O1–C2–C1	122.0 (3)	O1–C2–C3	117.9 (3)
C1–C2–C3	120.1 (3)	N1–C3–C5	126.6 (3)
N1–C3–C2	115.0 (3)	C5–C3–C2	118.4 (3)
O2–C4–N2	126.9 (3)	O2–C4–C4#1	122.4 (3)
N2–C4–C4#1	110.6 (3)	C3–C5–H5	109.5
C3–C5–H4	109.5	H5–C5–H4	109.5
C3–C5–H6	109.5	H5–C5–H6	109.5
H4–C5–H6	109.5		

### Analytical data

The data of CHN and metal contents of the complexes are presented in Table 3. The values confirm mononuclear complexes: [VO(OBH–H)_2_]·H_2_O, [Co(OBH)_2_Cl]Cl·½EtOH, [Cu(OBH)_2_Cl_2_]·2H_2_O, [Zn(OBH–H)_2_], [Zr(OBH)Cl_4_]·2H_2_O and binuclear complexes: [Ni_2_(OBH)Cl_4_]·H_2_O·EtOH and [Pd_2_(OBH)(H_2_O)_2_Cl_4_]·2H_2_O. All complexes are colored, solid and stable towards air and moisture at room temperature. They have high melting points and are insoluble in most common organic solvents and completely soluble in DMSO. The molar conductance values (Table 3) of 10^−3^ mol L^−1^ DMSO solution proved the non-electrolytic nature. The measured value for the Co(II) complex supports the formation of [Co(OBH)_2_Cl]^+^Cl^−^·½EtOH [18].

**Table 3:** Elemental analysis and some physical properties of OBH and its complexes

Compound, empirical formula	M.W. (Found, m/e)	Color	M.P. (°C)	Λ (Ohm^−1^ cm^2^ mol^−1^)^a^	C % Calcd. (Found)	H % Calcd. (Found)	N % Calcd. (Found)	M % Calcd. (Found)
OBH C_10_H_14_N_4_O_4_	254.25 (255.30)	White	247–249	1.76	46.66 (47.04)	5.75 (5.55)	22.70 (22.34)	–
[Co(OBH)_2_Cl]Cl.½EtOH C_21_H_31_N_8_O_8.5_Cl_2_Co	661.395	Pale brown	>325	48.0	38.13 (38.13)	4.72 (4.94)	16.94 (16.68)	8.91 (8.63)
[Zr(OBH)Cl_4_]·2H_2_O C_10_H_18_N_4_O_6_Cl_4_Zr	523.33 (523.4)	Pale orange	>325	20.10	22.95 (22.63)	3.47 (3.87)	10.70 (10.79)	17.70 (17.20)
[Zn(OBH–H)_2_] C_20_H_26_N_8_O_8_Zn	571.84	Yellowish white	>325	2.62	42.09 (42.49)	4.58 (5.08)	19.59 (19.39)	
[VO(OBH–H)_2_]·H_2_O C_20_H_26_N_8_O_10_V	591.86	Brown	293	9.74	40.58 (39.99)	4.77 (4.98)	18.93 (18.28)	8.45 (7.93)
[Cu(OBH)_2_Cl_2_]·2H_2_O C_20_H_32_N_8_O_10_Cl_2_Cu	678.99	Yellowish green	>325	22.50	35.37 (35.35)	4.45 (4.58)	16.50 (16.07)	9.34 (9.08)
[Ni_2_(OBH)Cl_4_]·H_2_O·EtOH C_20_H_22_N_4_O_6_Cl_4_Ni_2_	577.62 (371.7)^b^	Reddish brown	>325	37.30	24.95 (24.53)	3.84 (4.11)	9.70 (9.39)	20.34 (20.54)
[Pd_2_(OBH)(H_2_O)_2_Cl_4_]·2H_2_O C_10_H_22_N_4_O_4_Cl_4_Pd_2_	618.18 (620.30)	Brown	>325	24.48	20.63 (20.96)	3.81 (4.20)	9.62 (9.39)	

^a^Molar conductance values for 0.001 mol L^−1^ DMSO solution

^b^The value represents Ni(OBH)Cl·½EtOH

### IR and NMR (^1^H and ^13^C) spectra

**Fig. 1:**
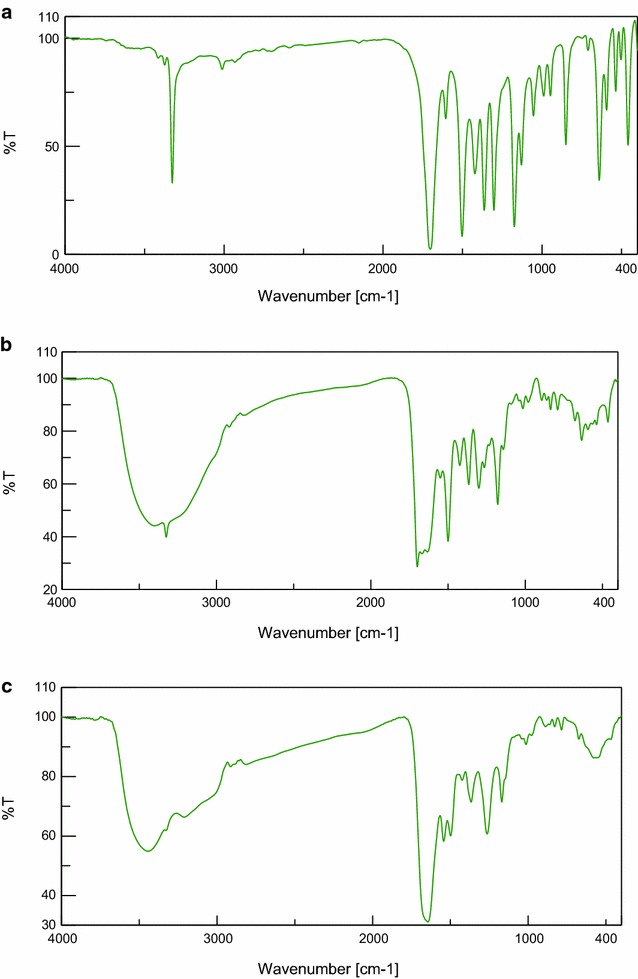
IR spectra of OBH (**a**); [Ni_2_(OBH)Cl_4_]·H_2_O·EtOH (**b**) and [Pd_2_(OBH)Cl_4_]·6H_2_O (**c**)

OBH showed the characteristic bands for ν(NH), ν(C=O) [ketonic and amidic] and ν(C=N) vibrations at 3325, 1701 and 1605, respectively in its IR spectrum (Fig. 1a). Inspections of the IR spectral data of the complexes, Table 4, three modes are suggested. The ^1^H NMR spectrum showed the NH and CH_3_ protons at 11.924 (s, 2H) and 2.129 (s, 6H) ppm, respectively. On the other hand, the ^13^C NMR spectrum have multiple peaks corresponding to (C=O)_ketoni_, (C=O)_amidic_, C=N and CH3 groups at 196.65, 167.58, 148.81 and 23.90 ppm.

**Table 4:** IR band assignments of OBH and its complexes

Compound	ν(NH)	ν(C=O)	ν(C=N)	ν(C=N)^a^	ν(C–O)	ν(M–O)	ν(M–N)	Observations
OBH	3325 (s)	1701 (s)	1605 (m)	–	–	–	–	
[Co(OBH)_2_Cl]Cl.½EtOH	3325 (m)	1699 (s)	1612 (w)	–	–	464 (m)	–	3415 (br) for EtOH
[Zr(OBH)Cl_4_]·2H_2_O	3326 (vbr)^a^	1686 (m)	1585 (m)	–	–	495 (br)	–	
[Zn(OBH–H)_2_]	–	1699 (s)	1605 (w)	1550 (w)	1140 (w)	463 (s)	–	
[VO(OBH–H)_2_]·H_2_O	–	1696 (s)	1608 (w)	1552 (w)	1142 (w)	463 (m)	–	3412 (br) for H_2_O; ν(V=O) at 961
[Cu(OBH)_2_Cl_2_]·2H_2_O	3325 (m)	1701 (s)	1610 (br)	–	–	464 (m)	–	3415 (br) for H_2_O
[Ni_2_(OBH)Cl_4_]·H_2_O·EtOH	3324	1676 (br)	1552 (sh)	–	–	464 (m)	539	3389 (br) for H_2_O
[Pd_2_(OBH)(H_2_O)_2_Cl_4_]·2H_2_O	3327 (w)	1644	1542	–	–	467	542	3441 (br) for H_2_O

^a^The value for NH and H_2_O

In the first mode, OBH acts as a neutral bidentate ligand in [Co(OBH)_2_Cl]Cl·½EtOH (Structure 2), [Cu(OBH)_2_Cl_2_]·2H_2_O and [Zr(OBH)Cl_4_]·2H_2_O coordinating through the two amidic carbonyl groups based on the following observations: the υ(C=O) band observed at 1701 cm^−1^ in ligand spectrum was shifted to 1686–1699 cm^−1^ in complexes having little intensity indicating that the two amidic carbonyl groups (C=O_amidic_) participated in bonding while the other two carbonyl (C=O_ketonic_) still at the same position. The new band at 464–495 cm^−1^ is due to υ(M–O) vibration [19]. The υ(C=N) at 1605 cm^−1^ appeared very weak, less intensity with little shift to higher wavenumber in the Co(II) and Cu(II) complexes and to lower wavenumber in the Zr(IV) complex (1585 cm^−1^).

**Structure 2:**
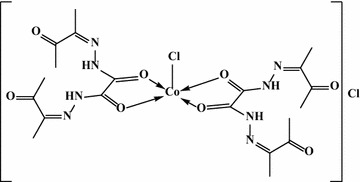
Structure of [Co(OBH)_2_Cl]Cl·½EtOH

In the second mode, OBH acts as a mononegative bidentate in Zn^2+^ and VO^2+^ complexes coordinating through the two amidic carbonyl (enolic form), from each ligand molecule. The shift of υ(C=O) to lower or higher wavenumbers with appearance of υ(C=N)*, υ(C–O) (due to enolization of one amidic group) [20] and υ(M–O) at 1550, 1140 and 463 cm^−1^ indicates the participation of carbonyl group in bonding. In the VO^2+^ complex, the band observed at 3412 cm^−1^ is attributed to hydrated water [21] and absence of sulfate bands indicates enol type of complexes. The ^1^H NMR spectrum of [Zn(OBH–H)_2_] showed splitting of NH signal as a result of conversion of one of NHC=O to N=C–OH and the existence of the others without participation (Structure 3). The signals of CH_3_ protons appeared at the same position as in ligand spectrum. In its ^13^C NMR, peaks of both ketonic and amidic groups still at the same position with appearance of a new one at 166.21 ppm although one of the C=O_amidic_ changed to enol form. Also, the appearance of C=N as doublet peak in 149.44–148.44 ppm range confirming enolization. In the ^13^C NMR spectrum of VO^2+^ complex, the peaks at 172.50, 168.44–167.47, 149.60–148.86, 124.68 and 24.98–24.30 ppm are due to (C=O)_ketonic_, (C=O)_amidic_ free, (C=O)_amidic_ bonded, (C=N), (C=N)*, (C–O) and CH_3_, respectively. The appearance of (C=N)* (due to conversion of NHC=O to N*=C–O) and (C–O) peaks confirm enolization process (Table 5).

**Structure 3:**
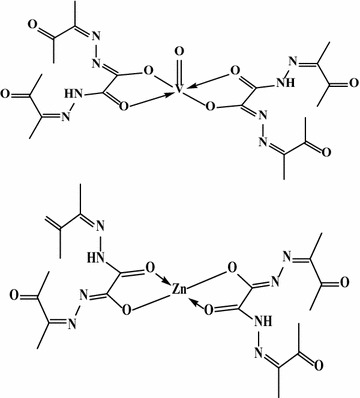
Structures of VO^2+^ and Zn^2+^ complexes

**Table 5:** ^1^H and ^13^C NMR signals of OBH and its diamagnetic complexes

Compound	NH	CH_3_	^13^C signals
OBH	11.924 (s, 2H)	2.129 (s, 6H)	196.65 (C=O)_ketonic_ 167.58; (C=O)_amidic_ 148.81 (C=N) 23.90 (CH_3_)
[Zn(OBH–H)_2_]	11.781 (s, 1H) 11.565 (s, 1H)	2.371 (s, 3H) 2.118 (s, 3H)	196.68 (C=O)_ketonic_ 168.02 (C=O)_amidic_ free 166.21 (C=O)_amidic_ bonded 149.44 (C=N), (C=N)* 23.94 (CH_3_)
[VO(OBH-H)_2_]·H_2_O	11.769 (s, 1H)	2.087 (s, 3H) 2.053 (s, 3H)	197.12 (C=O)_ketonic_ 172.50 (C=O)_amidic_ (free) 168.44–167.47 (C=O)_amidic_ (bonded) 149.60–148.86 (C=N), (C=N)* 124.68 (C–O) 24.68; 24.98 (CH_3_)
[Pd_2_(OBH)(H_2_O)_2_Cl_4_]·2H_2_O	11.766 (s, 1H) 11.566 (s, 1H)	2.290 (s, 6H)	196.75 (C=O)_ketonic_ 167.98; (C=O)_amidic_ 148.88 (C=N) 22.90 (CH_3_)

*New azomethine group as a result of enolization

The third mode confirmed neutral tetradentate but with two metal ions in [Ni_2_(OBH)Cl_4_]·H_2_O·EtOH (Structure 4) and [Pd_2_(OBH)(H_2_O)_2_Cl_4_]·2H_2_O (Fig. 1b, c). The coordination sites are two azomethine nitrogens of the hydrazone moiety and two carbonyl groups of amidic moiety; each two donors chelated one metal ion. The shift of υ(C=N) to 1542 cm^−1^ and υ(C=O)_amidic_ to 1644 in the Pd(II) complex and to 1552 and 1676 in the Ni(II) complex together with appearance of υ(M–N) [22] and υ(M–O) bands at ~465 and ~540 cm^−1^, respectively. In the Ni(II) complex, the band of carbonyl groups splitted to two at 1697 and 1676 cm^−1^; the first is due to ketonic group which is not participated in bonding. The NH band appeared very weak in Ni(II) complex and very broad in Pd(II) complex. Finally, the band at 3389 or 3441 cm^−1^ in Ni(II) or Pd(II) complex is due to hydrated water or ethanol.

**Structure 4:**
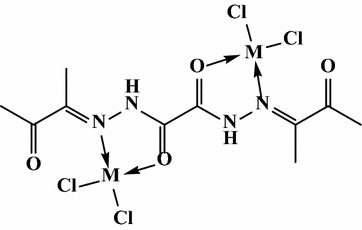
Structure of [Ni_2_(OBH)Cl_4_]·H_2_O·EtOH

### Mass spectra

The data of FAB-mass spectra of OBH and some of its complexes are shown in Table 3. The mass spectrum of OBH showed the molecular ion peak (base peak) at m/z = 255.30 (Calcd. 254.25) corresponding to C_10_H_14_N_4_O_4_. The peaks shown at 212.2, 190.2, 130.2 and 73.1 are due to C_8_H_11_N_4_O_3_, C_7_H_11_N_3_O_3_, C_4_H_5_N_3_O_2_ and C_2_NO_2_.

The mass spectrum of [Zr(OBH)Cl_4_]·2H_2_O exhibits m/z value of 523.5 (Calcd. 523.33) with 12 % intensity. The value corresponds to C_10_H_18_N_4_O_6_Cl_4_Zr. Multi-peaks were observed ending with a peak at 69.0 (78 % intensity) may corresponding to 6 C.

Moreover, the mass spectrum of [Ni_2_(OBH)Cl_4_]·H_2_O·EtOH has a value of 371.7 (the base peak) corresponding to Ni(OBH)Cl·½EtOH meaning that this species is highly stable. Multi peaks were observed ending with one at 128.9 (intensity 65 %) due to ZrO_2_.

### Magnetic moments and electronic spectra

The electronic spectral bands of the complexes as well as the magnetic moment values are presented in Table 6. The DMSO solutions of complexes have the same color as in the solid complexes. OBH exhibits one absorption band at 38,460 cm^−1^ collectively due to π → π* transitions of C=N, C=O_ketonic_ and C=O_amidic_ groups [23]. The broadness of the band may be due to existence of these groups in opposite sides. The two bands at 25,510 and 23,810 cm^−1^ in Cu(II) complex may be due to N → MCT and O → MCT [24]. The Ni(II) complex has only one band at 28,330 cm^−1^ due to N → MCT while Co(II) and Zr(IV) have also one band but at 23,320 and 22,830 cm^−1^, respectively, due to O → MCT.

**Table 6:** Magnetic moments, electronic spectra and molar extension coefficient of OBH and its complexes

Compound	μ_eff_ (BM)	Intraligand and charge transfer transition, cm^−1^ (*ɛ)	d–d transition cm^−1^ (*ɛ)	Proposed structure
OBH	–	38,460 (790)	–	
[Co(OBH)_2_Cl]Cl·½EtOH	2.51	37,450 (195.8); 23,320 (115.8)	15,250 (94)	Square-pyramid
[Zr(OBH)Cl_4_]·2H_2_O	–	36,495 (399); 22,830 (117.6)	–	Octahedral
[Zn(OBH–H)_2_]	–	37,450 (530); 35,335 (885)	–	Tetrahedral
[VO(OBH–H)_2_]·H_2_O	0.00	38,060; 35,040; 29.210		Square-pyramid
[Cu(OBH)_2_Cl_2_]·2H_2_O	1.45	39,840; 37,590; 25,510; 23,810 (350)	20,080	Octahedral
[Ni_2_(OBH)Cl_4_]·H_2_O·EtOH	1.36^a^	39,840; 37,590; 28,330	19,050	Square-planar + tetrahedral
[Pd_2_(OBH)(H_2_O)_2_Cl_4_]·2H_2_O	0.00	37,540; 28,470	21,500 (310)	Square-pyramid

*ɛ is the molar extension coefficient (mol^−1^L)

^a^The value per one nickel atom

[Co(OBH)_2_Cl]Cl·½EtOH (pale brown) has 2.51 BM magnetic moment which lies within the values reported for one unpaired electron of square-planar or square-pyramid Co(II) complexes [25] having dsp^2^ or dsp^3^ hybridization. Evidence is electronic spectrum which showed one band at 15,250 cm^−1^ with molar extension coefficient of 94 mol^−1^ L. The spectrum resembled the spectra of the five-coordinate Co(II) complexes [26] and the square-pyramid is the suggested geometry.

The magnetic moment value, for each atom, in [Ni_2_(OBH–2H)Cl_4_]·H_2_O·EtOH is 1.36 BM which is less than the normal values reported for tetrahedral or octahedral coordination containing two unpaired electrons. Its electronic spectrum showed a broad band at 19,050 cm^−1^ (ɛ = 180 mol^−1^ L) typical of a square-planar structure with some distortion [26] may be of tetrahedral; the anomalous magnetic value is consistent with mixed stereochemistry (square-planar + tetrahedral) around the two nickel ions [27]. On the other hand, the diamagnetic nature of [Pd_2_(OBH)(H_2_O)_2_Cl_4_]·2H_2_O proved the square-pyramid structure in which the metal is surrounded by NO donors, two chloro and one coordinated water. The bands at 37,540 and 28,470 cm^−1^ are attributed to charge transfer transitions, probably O → Pd transition [28].

The electronic spectrum of [Cu(OBH)_2_Cl_2_]·H_2_O exhibits one band with maximum at 20080 cm^−1^ assigned to the ^2^E_2g_ → ^2^T_2g_ transition in an octahedral geometry [29]. The band is broad due to the Jhan-Teller effect which enhances the distortion of the octahedral geometry generally important for odd number occupancy of the e_g_ level. The magnetic moment value (1.45 BM) was found lower than the values reported for the d^9^–system containing one unpaired electron (1.73–2.25 BM) suggesting interactions between the copper centers.

### Thermal analysis

The decomposition steps, the DTG maximum temperature and the removing species are shown in Table 7. The thermogram of [Co(OBH)_2_Cl]Cl·½EtOH showed three decomposition steps at mid- points of 60, 319 and 500 °C corresponding to the removal of ½Cl_2_ + ½EtOH (Found 6.36 %; Calcd. 8.84 %); C_16_H_24_N_4_O_6_Cl (Found 60.45 %; Calcd. 61.05 %) and C_4_H_4_N_2_ (Found 11.77 %; Calcd 12.11 %) leaving [CoO_4_N_2_] moiety (Found 21.58 %; Calcd. 22.82 %).

**Table 7:** Decomposition steps of the complexes based on the thermogravimetric data

Complex	DTG maximum temp. (°C)	Removing species	Weight loss % Found (Calcd)
[Co(OBH)_2_Cl]Cl·½EtOH	60	- ½Cl_2_ + ½EtOH	6.36 (8.84)
	319	- C_16_H_24_N_4_O_6_Cl	60.45 (61.05)
	500	- C_4_H_4_N_2_	11.77 (12.11)
	>500	[CoO_4_N_2_] (residue)	21.58 (22.82)
[Zr(OBH)Cl_4_]·2H_2_O	76	- (Cl_2_ + H_2_O)	16.12 (16.99)
	313	- (H_2_O + C_8_H_12_N_2_O_2_)	37.67 (35.58)
	449	- Cl_2_	12.44 (13.55)
	>500	C_2_N_2_O_2_Zr (residue)	30.15 (33.67)
[VO(OBH-H)_2_]·H_2_O	59	- H_2_O	3.65 (3.04)
	289	- C_16_H_24_N_4_O_6_	61.57 (62.24)
	405–590	- C_4_H_4_N_4_O_2_	25.56 (23.67)
	>600	V (residue)	7.45 (8.53)
[Cu(OBH)_2_Cl_2_]·2H_2_O	59	- 2H_2_O	4.29 (5.31)
	291	- C_16_H_24_N_4_O_6_	54.90 (54.26)
	374	- C_4_H_4_N_4_O_3_	25.56 (26.37)
	>400	CuO (residue)	12.30 (11.71)
[Ni_2_(OBH)Cl_4_]·H_2_O·EtOH	72	- (EtOH + H_2_O)	13.51 (11.08)
	368	- (Cl_2_ + C_8_H_12_N_2_O_2_)	40.20 (41.40)
	470	- Cl_2_	11.20 (12.27)
	550	- C_2_H_2_N_2_	8.00 (9.35)
	>600	2 NiO (residue)	27.09 (25.87)
[Pd_2_(OBH)(H_2_O)_2_Cl_4_]·2H_2_O	75	- 2H_2_O	6.47 (5.83)
	322	- 2H_2_O + 2Cl_2_ + C_4_H_12_	38.49 (38.48)

The TG curve of [VO(OBH-H)_2_]·H_2_O showed also three steps; the first (mid. point 56 °C) represents the removal of the outside water molecule (Found 3.65 %; Calcd 3.04 %); the second (mid. point 289 °C) represents the loss of C_16_H_24_N_4_O_6_ (Found 61.57 %; Calcd 62.24 %) and the third for the repulsion of C_4_H_4_N_4_O_2_ (Found 25.56 (Calcd. 23.67 %). The residue is vanadium metal (Found 7.45; Calcd 8.53 %).

[Cu(OBH)_2_Cl_2_]·2H_2_O thermogram showed decomposition steps ending with copper oxide at Temp. >400 °C. The decomposition showed the removal of the two hydrated water in the first step at mid. point of 59 °C. The other two steps were observed at 291 and 374 °C corresponding to the removal of C_16_H_24_N_4_O_6_ and C_4_H_4_N_4_O_3_, respectively, leaving CuO as a residue.

**Fig. 2:**
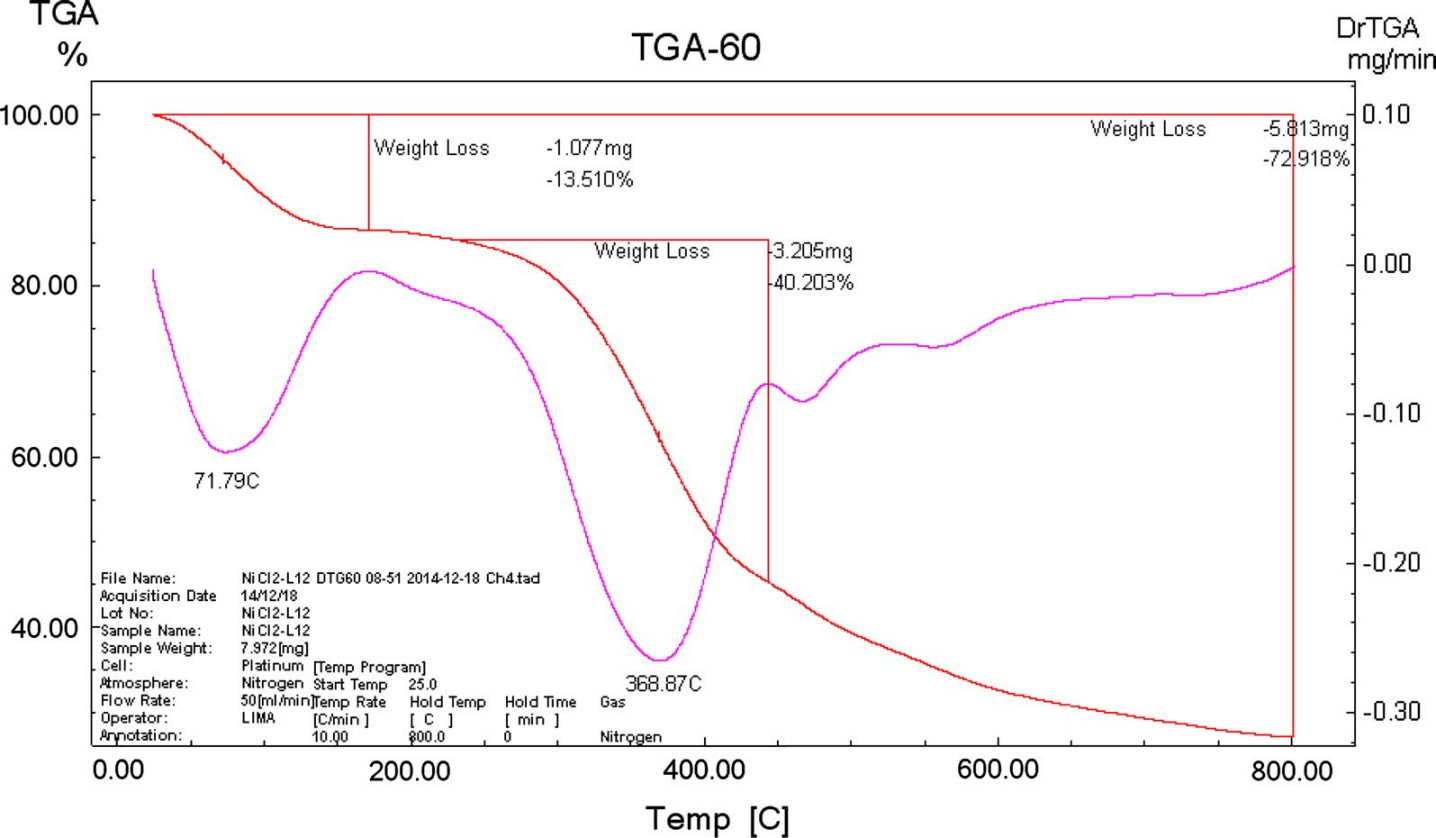
TGA thermogram of [Ni_2_(OBH)Cl_4_]·H_2_O·EtOH

The TG curve of [Ni_2_(OBH)Cl_4_]·H_2_O·EtOH showed four steps. The first at 72 °C is due to the removal of the outside water and EtOH (Found 13.51 %; Calcd. 11.08 %). The second step (368 °C) represents the loss of Cl_2_ + C_8_H_12_N_2_O_2_ (Found 40.20 %; Calcd 41.40 %). The third step represents the repulsion of Cl_2_ (Found 11.20 %; Calcd. 12.27 %). The fourth step (Found 8.00 %; Calcd. 9.35 %) corresponding to the removal of C_2_H_2_N_2_. The residue is 2NiO (Found 27.09 %; Calcd. 25.87 %) (Fig. 2).

**Fig. 3:**
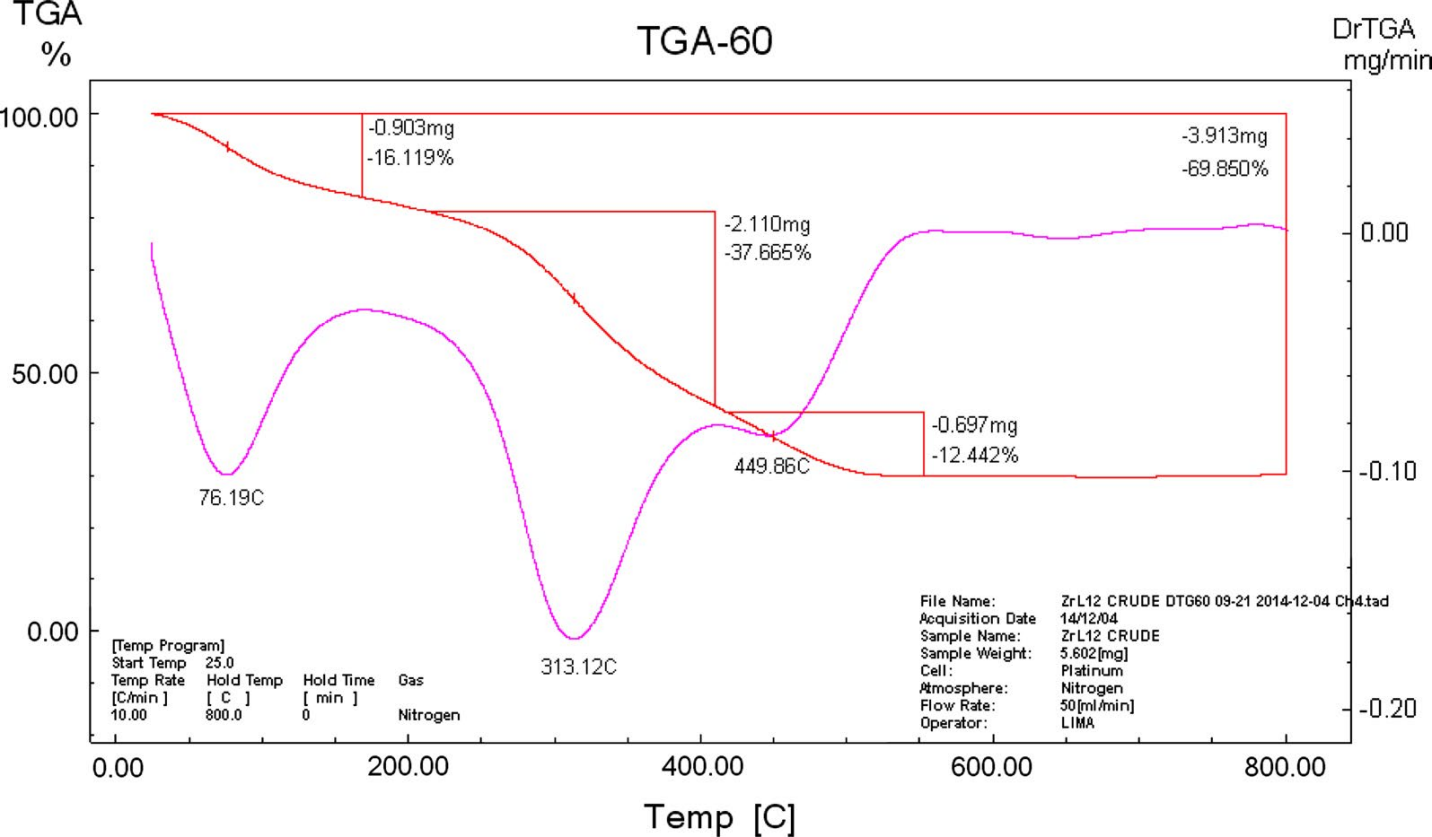
TGA thermogram of [Zr(OBH)Cl_4_]·2H_2_O

The thermogram of [Zr(OBH)Cl_4_]·2H_2_O has C_2_N_2_O_2_Zr as remaining residue above 500 °C with 30.15 % (Calcd. 33.67 %). The first three steps observed at mid. points of 76, 313 and 449 °C are corresponding to the removal of (Cl_2_ + H_2_O); (H_2_O + C_8_H_12_N_2_O_2_) and Cl_2_, respectively (Fig. 3).

The thermogram of [Pd_2_(OBH)(H_2_O)_2_Cl_4_]·2H_2_O showed two main steps at 75 and 322 °C due to the liberation of the outside water and 2H_2_O + 2Cl_2_ + C_4_H_12_, respectively. High residue  % was found over 500 °C.

### Molecular modeling

Trials to grow single crystals for the investigated complexes were failed. In order to calculate the molecular parameters, [Co(OBH)_2_Cl]Cl·½EtOH and [Ni_2_(OBH)Cl_4_]·H_2_O·EtOH (Structure 5) are chosen and their data are presented in Table 8. The bond lengths and the bond angles are shown in Additional file 1: Tables S1 and S2. It is obvious that the energy values obtained for [Ni_2_(OBH)Cl_4_]·H_2_O·EtOH are less than those of [Co(OBH)_2_Cl]Cl·½EtOH indicating that the presence of two metals stabilized the complex more than the mono metal lowering the energy. The dipole moment calculated for the Co(II) complex is 4.949 D proving the polar nature of the complex. The value of Ni(II) complex is 0.413 D indicating its non-polarity.

**Structure 5:**
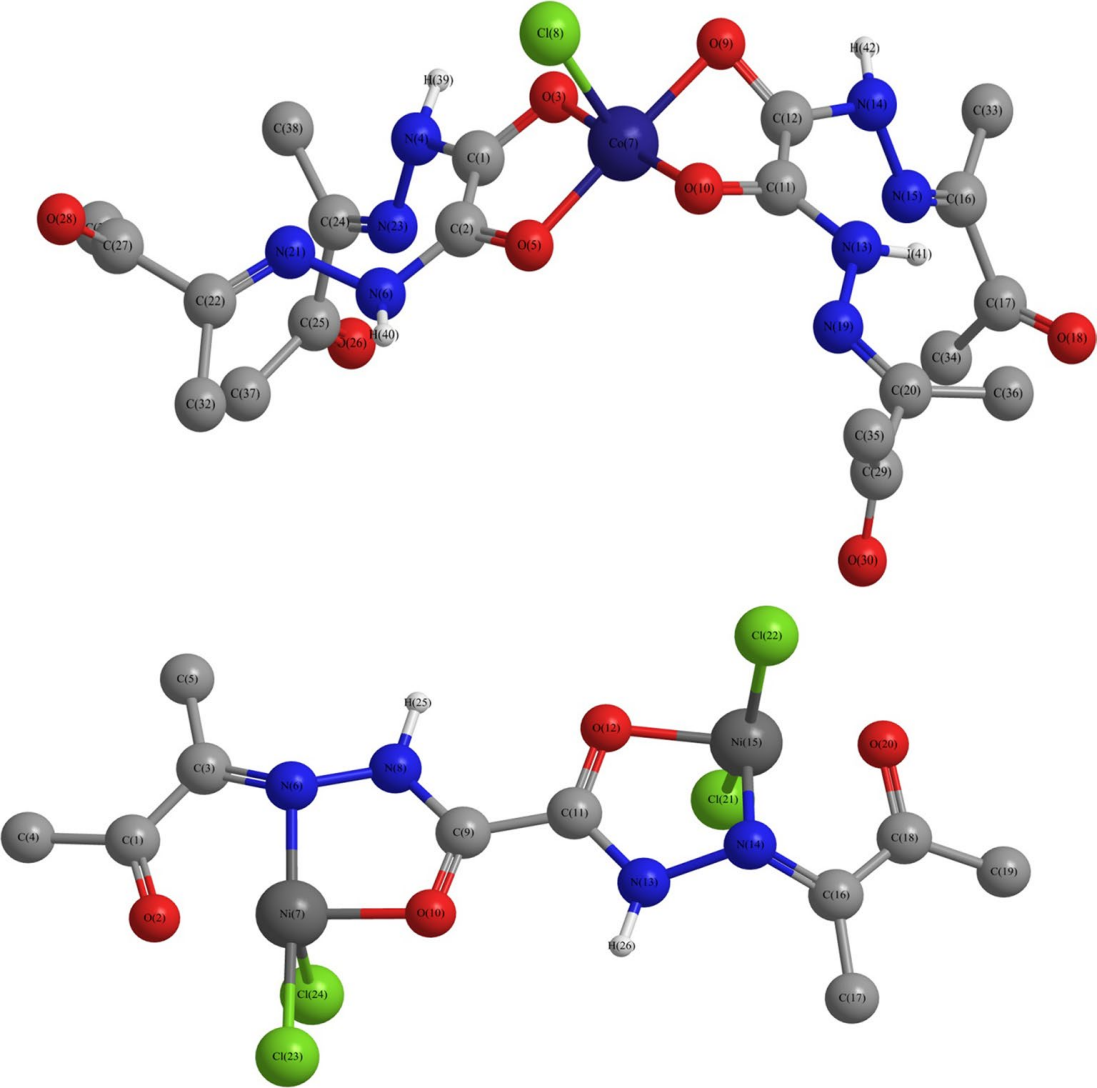
Molecular modeling of [Co(OBH)_2_Cl]Cl·½EtOH and [Ni_2_(OBH)Cl_4_]·H_2_O·EtOH

**Table 8:** Molecular parameters of the Co(II) and Ni(II) complexes

Parameter	[Co(OBH)_2_Cl]Cl·½EtOH	[Ni_2_(OBH)Cl_4_]·H_2_O·EtOH
Total energy (kcal/mol)	−174051.6072885	−151380.5229794
Total energy (a.u.)	−277.368857336	−241.240189251
Binding energy (kcal/mol)	−6649.8942255	−3610.0626914
Isolated atomic energy (kcal/mol)	−167401.7130630	−147770.4602880
Electronic energy (kcal/mol)	−1559859.4974432	−935598.9682580
Core–core interaction (kcal/mol)	1385807.8901548	784218.4452785
Heat of formation (kcal/mol)	−261.3762255	−159.9386914
Gradient (kcal/mol/Å)	53.4569309	42.0607388
Dipole (Debyes)	4.949	0.413

### Biological activity

The antimicrobial activity of the metal complexes depends on the following factors: the chelate effect, i.e., bidentate ligands show higher antimicrobial activity than monodentate; the nature of the ligands; the total charge of the complex: cationic > neutral > anionic; the nature of the counter ion and the nuclearity of the metal center: binuclear are more active than mononuclear ones. It depends more on the metal center itself than on the geometry around the metal ion.

The antimicrobial activities of OBH and its complexes are examined against *Bacillus, E. coli, Aspergillus, Penicillium and Fusarium* and the data are given in Table 9. The data showed that [Zr(OBH)Cl_4_]·2H_2_O has higher activity against all tested microorganisms except *E. coli*. The activity is highest and more with *Penicillium* (9 mm zone inhibition). The higher activity may be due the presence of non-ionizable chlorine and to the less planarity of the complex making it more lipophilic. Most compounds have high activity against *Fusarium*. [Cu(OBH)_2_Cl_2_]·2H_2_O has higher value against *Fusarium* (15 mm). Comparing these data with that of ampicillin and those obtained for different hydrazone complexes showed more or less activity [29, 30].

**Table 9:** Effect of ligand and its complexes on some microorganisms

Compound	*Bacillus*	*E. coli*	*Aspergillus*	*Penicillium*	*Fusarium*
OBH	Nil	2.0	Nil	Nil	Nil
[Co(OBH)_2_Cl]Cl·½EtOH	Nil	1.0	Nil	Nil	15
[Zr(OBH)Cl_4_]·2H_2_O	10	Nil	4.0	9.0	8.0
[Zn(OBH–H)_2_]	Nil	1.0	Nil	Nil	5.0
[Cu(OBH)_2_Cl_2_]·2H_2_O	Nil	2.0	Nil	5.0	Nil
[Ni_2_(OBH)Cl_4_]·H_2_O·EtOH	Nil	2.0	Nil	Nil	Nil
[Pd_2_(OBH)(H_2_O)_2_Cl_4_]·2H_2_O	Nil	Nil	Nil	Nil	5.0
[VO(OBH–H)_2_]·H_2_O	Nil	Nil	Nil	Nil	5.0
DMSO	Nil	Nil	Nil	Nil	Nil
Ampicillin	25	–	27	–	–
Gentamicin	–	48	20	25.9	–

Reading in diameter (mm)

## Conclusion

Oxalo bis(2,3-butanedionehydrazone) has been prepared and characterized by x-ray crystallography. It coordinates as neutral bidentate; mononegative bidentate and neutral tetradentate. The complexes have tetrahedral, square-planar and/or octahedral structures. The VO^2+^ and Co^2+^ complexes have square-pyramid structure. [Cu(OBH)_2_Cl_2_]·2H_2_O and [Ni_2_(OBH)Cl_4_]·H_2_O·EtOH decomposed to their oxides while [VO(OBH–H)_2_]·H_2_O to the metal. The energies from molecular modeling calculation is less in [Ni_2_(OBH)Cl_4_]·H_2_O·EtOH than those for [Co(OBH)_2_Cl]Cl·½EtOH indicating that the presence of two metals stabilized the complex more than the mono metal. The Co(II) complex is polar molecule while the Ni(II) is non-polar.

## Further materials

Crystallographic data for the structure reported in this paper have been deposited with Cambridge Crystallographic Data Center as supplementary publication CCDC-985982.

## Additional file

10.1186/s13065-015-0135-y Bond lengths and bond angles of the Ni(II) complex.** Table S2.** Bond lengths and bond angles of the Co(II) complex.
