# Supplementary material for: Spectral, thermal, molecular modeling and biological studies on mono- and binuclear complexes derived from oxalo bis(2,3-butanedionehydrazone)
Source: Chem Cent J. 2015 Dec 29;9:69. doi: 10.1186/s13065-015-0135-y (PMC4693428; doi:10.1186/s13065-015-0135-y)
Supplement: Supplementary file 1 — 10.1186/s13065-015-0135-y Bond lengths and bond angles of the Ni(II) complex. Table S2. Bond lengths and bond angles of the Co(II) complex. [file 13065_2015_135_MOESM1_ESM.docx]

**Table S1** Bond lengths and bond angles of the Ni(II) complex.

| Bond length | Å | Bond angle | ^o^ |
| --- | --- | --- | --- |
| C(18)-O(20) | 1.345 | O(20)-C(18)-C(19) | 115.936 |
| Ni(15)-Cl(22) | 2.191 | O(20)-C(18)-C(16) | 119.788 |
| Ni(15)-Cl(21) | 2.197 | C(19)-C(18)-C(16) | 124.258 |
| N(14)-C(16) | 1.426 | C(18)-C(16)-C(17) | 124.377 |
| N(14)-Ni(15) | 1.817 | C(18)-C(16)-N(14) | 114.258 |
| N(13)-H(26) | 1.002 | C(17)-C(16)-N(14) | 121.127 |
| N(13)-N(14) | 1.528 | Cl(22)-Ni(15)-l(21) | 158.493 |
| O(12)-Ni(15) | 1.855 | Cl(22)-Ni(15)-N(14) | 94.450 |
| C(11)-N(13) | 1.369 | Cl(22)-Ni(15)-O(12) | 89.487 |
| C(11)-O(12) | 1.291 | Cl(21)-Ni(15)-N(14) | 107.045 |
| Ni(15)-Cl(22) | 2.191 | Cl(21)-Ni(15)-O(12) | 88.456 |
| Ni(15)-Cl(21) | 2.197 | N(14)-Ni(15)-O(12) | 93.640 |
| N(8)-H(25) | 1.000 | C(16)-N(14)-Ni(15) | 100.380 |
| N(8)-C(9) | 1.372 | C(16)-N(14)-N(13) | 118.082 |
| Ni(7)-Cl(24) | 2.199 | Ni(15)-N(14)-N(13) | 96.059 |
| Ni(7)-Cl(23) | 2.206 | H(26)-N(13)-N(14) | 119.462 |
| Ni(7)-O(10) | 1.847 | H(26)-N(13)-C(11) | 120.938 |
| N(6)-N(8) | 1.434 | N(14)-N(13)-C(11) | 114.798 |
| N(6)-Ni(7) | 1.813 | Ni(15)-O(12)-C(11) | 103.889 |
| C(3)-N(6) | 1.411 | N(13)-C(11)-O(12) | 119.303 |
| C(18)-O(20) | 1.345 | N(13)-C(11)-C(9) | 122.071 |
| N(14)-C(16) | 1.426 | O(12)-C(11)-C(9) | 118.620 |
| N(14)-Ni(15) | 1.817 | C(9)-O(10)-Ni(7) | 105.370 |
| N(13)-H(26) | 1.002 | C(11)-C(9)-O(10) | 118.313 |
| N(13)-N(14) | 1.528 | C(11)-C(9)-N(8) | 121.922 |
| O(12)-Ni(15) | 1.855 | O(10)-C(9)-N(8) | 119.764 |
| C(11)-N(13) | 1.369 | H(25)-N(8)-C(9) | 121.205 |
| C(11)-O(12) | 1.291 | H(25)-N(8)-N(6) | 120.530 |
| Ni(15)-Cl(22) | 2.191 | C(9)-N(8)-N(6) | 113.675 |
| Ni(15)-Cl(21) | 2.197 | Cl(24)-Ni(7)-Cl(23) | 85.203 |
| N(14)-C(16) | 1.426 | Cl(24)-Ni(7)-O(10) | 91.940 |
| N(14)-Ni(15) | 1.817 | Cl(24)-Ni(7)-N(6) | 110.371 |
| O(12)-Ni(15) | 1.855 | Cl(23)-Ni(7)-O(10) | 87.543 |
| C(11)-N(13) | 1.369 | Cl(23)-Ni(7)-N(6) | 164.424 |

**Table S2** Bond lengths and bond angles of the Co(II) complex.

| **Bond length** | **Å** | **Bond angle** | **^o^** |
| --- | --- | --- | --- |
| C(29)-C(35) | 1.500 | C(35)-C(29)-O(30) | 123.985 |
| C(29)-O(30) | 1.213 | C(35)-C(29)-C(20) | 115.286 |
| C(27)-C(31) | 1.502 | O(30)-C(29)-C(20) | 120.717 |
| C(27)-O(28) | 1.213 | C(31)-C(27)-O(28) | 123.142 |
| C(25)-C(37) | 1.502 | C(31)-C(27)-C(22) | 116.212 |
| C(25)-O(26) | 1.213 | O(28)-C(27)-C(22) | 120.641 |
| C(24)-C(38) | 1.489 | C(37)-C(25)-O(26) | 123.372 |
| C(24)-C(25) | 1.510 | C(37)-C(25)-C(24) | 115.675 |
| N(23)-C(24) | 1.301 | O(26)-C(25)-C(24) | 120.948 |
| C(22)-C(32) | 1.490 | C(38)-C(24)-C(25) | 116.757 |
| C(22)-C(27) | 1.511 | C(38)-C(24)-N(23) | 127.033 |
| N(21)-C(22) | 1.302 | C(25)-C(24)-N(23) | 116.204 |
| C(20)-C(36) | 1.489 | C(24)-N(23)-N(4) | 121.928 |
| C(20)-C(29) | 1.512 | C(32)-C(22)-C(27) | 116.902 |
| N(19)-C(20) | 1.301 | C(32)-C(22)-N(21) | 126.856 |
| C(17)-C(34) | 1.501 | C(27)-C(22)-N(21) | 116.217 |
| C(17)-O(18) | 1.213 | C(22)-N(21)-N(6) | 122.303 |
| C(16)-C(33) | 1.489 | C(36)-C(20)-C(29) | 116.425 |
| C(16)-C(17) | 1.509 | C(36)-C(20)-N(19) | 127.241 |
| N(15)-C(16) | 1.302 | C(29)-C(20)-N(19) | 116.329 |
| N(14)-H(42) | 1.004 | C(20)-N(19)-N(13) | 121.607 |
| N(14)-N(15) | 1.391 | C(34)-C(17)-O(18) | 123.626 |
| N(13)-H(41) | 1.003 | C(34)-C(17)-C(16) | 115.278 |
| N(13)-N(19) | 1.395 | O(18)-C(17)-C(16) | 121.093 |
| C(12)-N(14) | 1.437 | C(33)-C(16)-C(17) | 116.787 |
| C(11)-C(12) | 1.483 | C(33)-C(16)-N(15) | 126.762 |
| C(11)-N(13) | 1.387 | C(17)-C(16)-N(15) | 116.445 |
| O(10)-C(11) | 1.297 | C(16)-N(15)-N(14) | 122.352 |
| O(9)-C(12) | 1.333 | H(42)-N(14)-N(15) | 113.767 |
| Co(7)-O(10) | 1.924 | H(42)-N(14)-C(12) | 113.208 |
| Co(7)-O(9) | 1.909 | N(15)-N(14)-C(12) | 112.933 |
| Co(7)-Cl(8) | 2.168 | H(41)-N(13)-N(19) | 116.625 |
| N(6)-H(40) | 1.002 | H(41)-N(13)-C(11) | 119.215 |
| N(6)-N(21) | 1.392 | N(19)-N(13)-C(11) | 119.389 |
| O(5)-Co(7) | 1.903 | N(14)-C(12)-C(11) | 125.101 |
| N(4)-H(39) | 1.001 | N(14)-C(12)-O(9) | 115.947 |
| N(4)-N(23) | 1.398 | C(11)-C(12)-O(9) | 115.978 |
| O(3)-Co(7) | 1.887 | C(12)-C(11)-N(13) | 123.859 |
| C(2)-N(6) | 1.414 | C(12)-C(11)-O(10) | 116.891 |
| C(2)-O(5) | 1.302 | N(13)-C(11)-O(10) | 119.208 |
| C(1)-N(4) | 1.424 | C(11)-O(10)-Co(7) | 94.694 |
| C(1)-O(3) | 1.311 | C(12)-O(9)-Co(7) | 88.941 |
| C(1)-C(2) | 1.441 | O(10)-Co(7)-O(9) | 88.730 |
|  |  | O(10)-Co(7)-Cl(8) | 89.629 |
|  |  | O(10)-Co(7)-O(5) | 89.111 |
|  |  | O(10)-Co(7)-O(3) | 177.511 |
